# Acute Water Supplementation Improved the Body Composition of Young Female Adults After Water Restriction of 12 h in Baoding, China: A Randomized Controlled Trial (RCT)

**DOI:** 10.3389/fnut.2022.880630

**Published:** 2022-06-20

**Authors:** Jianfen Zhang, Na Zhang, Songming Du, Shufang Liu, Guansheng Ma

**Affiliations:** ^1^Department of Nutrition and Food Hygiene, School of Public Health, Peking University, Beijing, China; ^2^Laboratory of Toxicological Research and Risk Assessment for Food Safety, Peking University, Beijing, China; ^3^Chinese Nutrition Society, Beijing, China; ^4^School of Public Health, Health Science Center, Hebei University, Baoding, China

**Keywords:** water restriction, water supplementation, rehydration, body composition, young adults

## Abstract

Insufficient intake of water may influence the balance of water in the human body. In this study, we explore the impacts of water supplementation on body composition indices among young adults after 12 h of water restriction, with the aim to determine the optimum volume of water for improving body water composition. A randomized controlled trial study was conducted among 64 young men and women in Baoding, China. After fasting overnight for 12 h, anthropometric measurements and urine and blood samples were collected as a baseline test at 8:00 a.m. of Day 2. Body composition was determined by measuring the ECW (extracellular water), ICW (intracellular water), and TBW (total body water) through bioelectrical impedance analysis (BIA). The participants were randomly divided into four groups, including water supplementation (WS) groups 1, 2, and 3, with 500, 200, and 100 mL of water, respectively, and a no water supplementation (NW) group. After 90 min, they were reassessed in a rehydration test (at 10:00 a.m. of Day 2). Repeated measurement ANOVA was used to assess the impact of water supplementation on body composition. Comparing the baseline and rehydration tests, interactions between time and volume were not significant among the men (all *p* > 0.05). Among women, a significant interaction was only found in ECW (*p* = 0.043), with TBW tending toward being significant (*p* = 0.055). Comparing the baseline and rehydration tests, the ECW in WS group 1, WS group 3, and the NW group all decreased (*p* = 0.028, *p* = 0.001, and *p* = 0.029), with reductions of 0.1, 0.3, and 0.2 kg, respectively; however, no significant decrease was observed in WS group 2 (*p* = 0.329). Furthermore, comparing the WS groups with the NW group in the rehydration test, the differences were not significant (*p* = 1.000, *p* = 1.000, and *p* = 0.288, respectively). Between men and women within groups, all of the body composition indices differed significantly, both at baseline and rehydration tests (all *p* < 0.05). Water supplementation led to changes in the distribution of the water content in young female adults in this study—but not men—after 12 h of water restriction, with no decrease in ECW. Finally, we found that 200 mL was the minimum volume capable of improving the water content distribution in participants in this study.

**Trial Registration:**

[www.chictr.org.cn], identifier [ChiCTR-IOR-17011568].

## Background

Water is an abundant compound in the human body, representing approximately 45–75% of the body mass (1, 2). It is well-known that the total body water (TBW), consisting of intracellular water (ICW) and extracellular water (ECW)—which comprise 65 and 35% of the TBW, respectively—is constantly maintained. In humans, water is mainly secured through three routes: total drinking fluids, water from food, and metabolic water. Meanwhile, the main output of water is by urine, through excretion by the kidneys, and in sweat evaporated from the skin (3). Other routes of water loss include breathing and fecal excretion (4). Generally, there is a dynamic balance in water intake and output, which is called the hydration status of the human body. Insufficient water intake may lead to dehydration, which could degrade cognition (5, 6). Therefore, it is crucial to take in adequate water to maintain the optimal hydration status in human beings.

Studies investigating dehydration have shown that there are two types of dehydration, defined as intracellular dehydration and extracellular dehydration (7, 8). It has been revealed that cellular hydration plays a physiological role in regulating cell function. Despite the adverse effects of dehydration on human health, overhydration also needs to be avoided, which may also impede health (9–11). Therefore, analysis of the specific proportions of water in each section of the human body (e.g., TBW) is a regular diagnostic practice in some research areas. A series of studies have demonstrated that body composition information is used in various fields of medicine; for example, to supplement methods for distinguishing patients at risk of post-operative morbidity (12), to verify the effects of dietary supplements (10), and for monitoring seasonal changes in the nutritional status and body composition of athletes (13). As for patients, higher ECW/TBW (extracellular water to total body water ratio), ECW, ICW, ECW/ICW (extracellular water to intracellular water ratio), fat-free mass (FFM), and TBW/FFM have been linked with various health outcomes, including lower odds of frailty, lower survival rates, and higher level of albuminuria (14–18). Furthermore, in the COVID-19 era, the highest ECW/TBW has been observed in older adults with osteosarcopenic adiposity syndrome (OSA), indicating a heightened inflammatory state (19). As has been described previously, body composition data are useful for assessing diverse disease conditions; furthermore, they have been associated with physiological status among healthy people (20–25).

Although some studies have evaluated the associations between body composition and health, only several studies thus far have investigated the relationship between hydration status and body composition, with inconsistent results. Studies have shown that total drinking fluids are associated with TBW, confirming the relationship between hydration status and body composition and that TBW/BW decreases with increasing dehydration among children (26–28). In addition, the impairments due to dehydration or water restriction on body composition have been evaluated clearly in studies conducted among people or animals (29–31). To our knowledge, only a few studies have analyzed the correlation between the body composition and water supplementation, in which the researchers did not obtain similar results (32, 33). Hence, more studies are needed for this issue.

Regarding the technologies for assessing the body composition of people, several methods have been used, including bioelectrical impedance analysis (BIA), dual-energy X-ray absorptiometry (DXA), quantitative magnetic resonance (QMR) EchoMRI systems, air displacement plethysmography (ADP), and magnetic resonance imaging (MRI) (34, 35). DXA is a mature technology for examining the mineral content of bone and body composition with high validity and stability (36), while the deuterium dilution technique and bromate sodium method have been recognized as reference procedures for determining the TBW and ECW, respectively (37). Nevertheless, some of these techniques and methods are expensive, invasive, and/or complicated, making them difficult to apply when considering large samples at the population level. Meanwhile, due to these revealed limitations (38), BIA has been considered as an alternative method for the quantification of water content and other components of body composition, thanks to its features of being portable, low-cost, rapid, non-invasive, safe for repeated measures, simple, and reproducible (39). A series of studies has demonstrated the validity of BIA for measuring the ICW, ECW, TBW, and FFM among healthy adults, children, and athletes with high validity (40–48). In free-living conditions, it offers a potential means to assess changes in distribution in body composition (e.g., ICW, ECW, and TBW).

Through this study, we aim to evaluate the following hypothesis: Acute water supplementation can lead to changes in some aspects of the body composition distribution, and different volumes of water may lead to different changes in the distribution of body composition among young Chinese men and women after 12 h of water restriction.

## Materials and Methods

### Study Design

A randomized controlled trial was implemented, which included two study days.

### Study Participants

Participants in a healthy state and aged 18–23 years were included in this study. The following exclusion criteria were used: participants with (a) habitual smoking, (b) high alcohol consumption (>20 g/day), (c) high-level caffeine consumption (>250 mg/day), (d) chronic diseases, and (e) other diseases (49). The participants were recruited in three ways: First, an advertisement was put up in the publicity window at a college; second, the researchers held several informative meetings, to which all college students could come if they wanted to learn about our research; third, notices of recruitment were sent through the instant messaging apps, including WeChat and QQ (Tencent Holdings Ltd., Shenzhen, China). For the selected participants, we arranged further medical examination, to exclude those with diseases. Finally, 64 participants were recruited for our study, half men and half women.

### Sample Size Calculation

In one study implemented among young men, the TBW under optimal hydration and dehydration statuses were 48.8 ± 7.5 and 45.4 ± 7.3 L, respectively (50). Then, the sample size was calculated using the PASS 11.0 (NCSS, LLC, Kaysville, UT) software, with α set at 0.05 (*p* < 0.05, two-tailed) and power (1-Beta) at 0.90. In addition, a 10% drop-out rate was considered. We found that, in total, 64 participants were required.

### Study Design and Procedure

A randomized controlled study design was implemented among the young men and women, which lasted 2 days.

**FIGURE 1:**
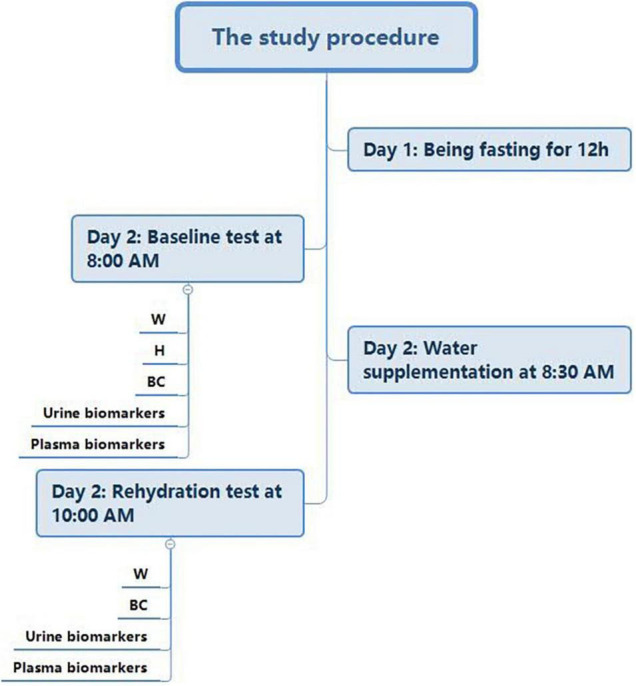
Flowchart of the study procedure (Note: H, height; W, weight; BC, body composition).

On Day 1, all of the participants were instructed to fast for 12 h (from 8:00 p.m. of Day 1 to 8:00 a.m. of Day 2). During the fasting period, they were not allowed to have any food or drinks. On the morning of Day 2 (8:00 a.m.), the baseline test, which included the measurement of anthropometric indices (height, weight, and body composition) and urine and blood samples (to assess the osmolality of urine and plasma, as well as urine specific gravity; USG), was conducted by trained investigators. Then, the participants were randomly separated into four groups: WS groups 1, 2, and 3 (administered 500, 200, and 100 mL of purified water, respectively) and the NW group (with no water). Each group included 8 men and 8 women. At 8:30 a.m., the participants in WS groups were instructed to drink the water supplied to them within no longer than 10 min, while the participants in NW group were not allowed to drink any fluids. The interval of water supplementation was 90 min. At 10:00 a.m., participants were reassessed through a rehydration test. The study procedure is shown in Figure 1.

### Temperature and Humidity of the Environment

The participants were college students in Hebei province, China, and were not allowed to engage in intense activities 1 week before and during the study days. The places that they were allowed access to included their dormitories and classrooms, allowing the micro-climate both outdoors and indoors to be recorded. Therefore, the temperature and humidity of the indoors and outdoors were recorded at three-time points—namely, 10:00 a.m., 2:00 p.m., and 8:00 p.m.—over the 2 days (WRB-1-H2, Exasace, Zhengzhou, China). The mean temperature and humidity indoors were 22.8°C and 72%, respectively, while those outdoors were 19.3°C and 89%, respectively.

### Anthropometry

Weight and height were determined twice, to the nearest 0.1 kg and 0.1 cm, respectively, by trained investigators using standard procedures (HDM-300; Huaju, Zhejiang, China). BMI was calculated as weight (kg)/height squared (m^2^).

Body composition was determined a single time by trained investigators using a bioelectrical impedance analyzer (Inbody 720; Inbody; Seoul, South Korea). Based on the four component (4C) body composition model, the instrument uses eight-point contact electrodes (two thumb electrodes, two palm electrodes, two sole electrodes, and two heel electrodes, with the surface area all bigger than 4 cm^2^) to measure 30 impedance values in five segments (left and right arms and legs, trunk: RA, LA, TR, RL, and LL) at six different frequencies (1, 5, 50, 250, 500, and 1,000 kHz). The Inbody 720 uses different high- and low-frequency conditions to measure the intracellular and extracellular water, to accurately analyze the total water content. The BIA measurements were performed in the morning of Day 2 (i.e., at 8:00 AM) after overnight fasting for 12 h, and after water supplementation for 90 min (i.e., at 10:30 AM) in the laboratory, with a room temperature of 24°C. The investigators checked the BIA before the morning of Day 2, to ensure system self-test and calibration: At least two investigators were measured by the BIA twice to ensure the normal operation of the BIA. Participants were asked to wear light clothing (e.g., shorts and short sleeves) and bare feet and to ensure that no metals were present in their clothing. Before standing on the BIA platform, participants were asked to urinate and instructed to clean their hands and feet with alcohol, and then to wait on the chair nearby with bare feet for at least 5 min. After the trained investigator had input their information, including their BMI, age, gender, and height, into the BIA, the participants were instructed to stand on the two-foot electrodes of the BIA, with the rear sole first and then the front sole completely coinciding with the electrode; to hold the two hand electrodes, with their thumb and palm in tight contact with the electrode; and to keep the angle between their trunk and arms at 15° for 2 min. They were asked to stand still with calm breathing and to stay relaxed over the 2 min. Then, the ICW, ECW, TBW, and FFM of the participants were measured separately by the BIA, and the data were stored in its database. When the body composition measurement of each participant had finished, it was printed immediately for the investigators to record. The investigators checked the impedance data (Z and Xc) for RA, LA, TR, RL, and LL on the report, which declined directly from 1 kHz to 1,000 kHz, to ensure the success of measurement. Moreover, after collection of the body composition data for all of the participants, the database was copied onto a USB drive, to ensure data integrity and security.

We collected urine samples from the participants during their water restriction between 8:00 p.m. of Day 1 to 8:00 a.m. of Day 2. Furthermore, they were asked to drink as usual before fasting overnight. The color of each urine sample was checked carefully by the researchers, to confirm the corresponding hydration status, and almost all participants showed optimal hydration status.

### Thirst, Urine, and Plasma Biomarkers

Thirst was explored using the 10 cm line (51), which has been described in our previous study (5). Participants were provided with a 10 cm line and instructed to inscribe a mark anywhere on the line according to their feelings, with two extreme answers (“not at all” and “extremely”) at opposite ends of the line. Higher scores indicated that the participants were thirstier. The urine samples, including the first-morning urine and the urine after water supplementation, were collected and stored at +4°C in a special refrigerator before any measurements. After collection and centrifugation, all blood samples were retained for the following measurements. The osmolality of urine and plasma were determined using the freezing point method (SMC 30C; Tianhe, Tianjin, China). The measurement of USG (urine specific gravity) was carried out through the method of uric dry chemistry (H-800; Dirui, Changchun, China).

### Statistics

Results are expressed as means and standard deviations, numbers, and percentages (the hydration status). The normality of quantitative variables was assessed using quantile plots and the Shapiro–Wilk tests. One-way ANOVA and the Bonferroni correction method were used to explore the differences in data that were normally distributed among the four groups. Chi-squared tests were used to examine the differences in the hydration status of individuals. Differences in variables between men and women within each group were analyzed using Student’s *t*-test, both in the baseline test and rehydration test. The significance of differences in the variables between the baseline and rehydration tests was analyzed using Student’s paired *t*-tests. Repeated measurement ANOVA was used to assess the effects of water supplementation on body composition. All statistical analyses were performed using the SPSS 21.0 software (IBM Corp., Armonk, NY, United States). A *p*-value less than 0.05 was considered to indicate statistical significance. The Bonferroni correction for multiple tests was employed, and the alpha level was set at 0.008 (0.05/6 comparisons).

## Results

### Characteristics of Participants

In total, 64 participants were recruited and completed the study, with a completion rate of 100%. As shown in Supplementary Table 1, no significant differences were observed in the characteristics among the four groups (*p* > 0.05).

### Water Supplementation Effects on Thirst and Hydration Status

As shown in Supplementary Table 2, there was a significant main effect of time (*p* = 0.037), but not volume (*p* = 0.797), and a significant interaction between time and volume was found for thirst (*p* = 0.001). When comparing the rehydration and baseline tests, for thirst, significant reductions were found in WS groups 1 and 2 (*p* = 0.003 and *p* = 0.042, respectively), with no significant decrease in WS group 3 (*p* = 0.529) and an increase in the NW group (*p* = 0.039). *Post hoc* analysis revealed that significant differences were found between the NW group and WS groups 1 (*p* < 0.001) and 2 (*p* = 0.007), but not with WS group 3 (*p* = 0.892). For urine osmolality, there was significant effect of time (*p* < 0.001), but not volume (*p* = 0.822), and a significant interaction between time and volume was found for urine osmolality (*p* < 0.001). The t-tests showed that WS groups 1 and 2 decreased significantly (*p* < 0.001 and *p* = 0.004, respectively), the NW group increased significantly (*p* < 0.001), and the decrease in WS group 3 was not significant (*p* = 0.596). In terms of hydration status, there were significant improvements in WS groups 1 and 2 (*p* < 0.001 and *p* = 0.004, respectively). *Post hoc* analysis demonstrated that significant differences were found between the NW group and WS groups 1, 2, and 3 (*p* < 0.001, *p* < 0.001, and *p* = 0.045, respectively); however, no significant differences were found for thirst and hydration status between WS groups 1 and 2 (*p* = 1.000 and *p* = 0.090, respectively). Furthermore, there were no significant interactions between time and volume in any of the plasma biomarkers, including plasma osmolality (all *p* > 0.05).

### Water Supplementation Effects on Body Composition

As shown in Table 1, in the baseline test, the body composition indices did not differ significantly among the four groups (all *p* > 0.05), except for TBW/BW (*p* = 0.041). The *post hoc* analysis demonstrated that no significant differences were found between the WS groups and the NW group (*p* = 0.085, *p* = 1.000, and *p* = 0.098, respectively). Meanwhile, during the rehydration test, no significant differences were found in any of the body composition indices considered in this study among the four groups (all *p* > 0.05). When comparing the baseline and rehydration tests, no significant interactions between time and volume were found among the four groups (all *p* > 0.05), as shown in Table 1. Furthermore, the main effects of time and volume were not statistically significant in ICW, ICW/TBW, ECW, ECW/TBW, ECW/ICW, TBW, TBW/BW, and TBW/FFM (all *p* > 0.05).

**TABLE 1:** Body composition indices of participants.

Total	Baseline test				Rehydration test				*p*_*interaction*_
	NW group (*n* = 16)	WS group 1 (*n* = 16)	WS group 2 (*n* = 16)	WS group 3 (*n* = 16)	NW group (*n* = 16)	WS group 1 (*n* = 16)	WS group 2 (*n* = 16)	WS group 3 (*n* = 16)	
ICW (kg)	19.4 ± 3.5	21.4 ± 5.3	21.2 ± 4.2	21.1 ± 2.8	19.1 ± 3.7	21.1 ± 5.4	21.0 ± 4.2	20.8 ± 3.7	0.921
ICW/TBW (%)	62.5 ± 0.6	62.4 ± 0.6	62.4 ± 0.7	62.4 ± 0.7	62.3 ± 0.6	62.3 ± 0.5	62.3 ± 0.7	62.3 ± 0.7	0.796
ECW (kg)	11.7 ± 2.0	12.8 ± 3.1	12.8 ± 2.3	12.7 ± 1.6	11.6 ± 2.1	12.7 ± 3.1	12.7 ± 2.3	12.6 ± 1.7	0.909
ECW/TBW (%)	37.6 ± 0.6	37.6 ± 0.6	37.6 ± 0.7	37.6 ± 0.7	37.7 ± 0.6	37.7 ± 0.5	37.7 ± 0.8	37.7 ± 0.7	0.796
ECW/ICW (%)	60.2 ± 1.5	60.2 ± 1.5	60.3 ± 1.8	60.3 ± 1.8	60.6 ± 1.6	60.5 ± 14	60.6 ± 2.0	60.5 ± 1.8	0.801
TBW (kg)	31.1 ± 5.5	34.2 ± 8.5	34.0 ± 6.4	33.8 ± 4.3	30.7 ± 5.8	33.8 ± 8.5	33.7 ± 6.4	33.4 ± 4.7	0.918
TBW/BW (%)	52.1 ± 4.3	56.4 ± 5.7	54.0 ± 4.5	56.3 ± 4.9	51.8 ± 4.5	55.7 ± 5.7	53.6 ± 4.7	55.8 ± 5.3	0.643
TBW/FFM (%)	73.9 ± 2.6	73.3 ± 0.2	73.3 ± 0.3	73.3 ± 0.2	74.0 ± 2.6	73.3 ± 0.2	73.3 ± 0.3	74.0 ± 2.6	0.819

*Values are shown as the mean ± standard deviation (SD). No significant differences were found in ICW, ICW/TBW, ECW, ECW/TBW, ECW/ICW, TBW, TBW/BW, and TBW/FFM (both in the baseline and rehydration test all p > 0.05).*

It can be seen, from Table 2, that when comparing the baseline and rehydration tests, the ICW, ICW/TBW, ECW, ECW/TBW, ECW/ICW, TBW, TBW/BW, and TBW/FFM among the men in the four groups did not differ significantly. Furthermore, comparing baseline and rehydration tests, no significant interactions were found in any of the body composition indices (all *p* > 0.05). The main effects of time and volume were not statistically significant in any of the body composition indices (all *p* > 0.05).

**TABLE 2:** Body composition indices of men and women.

Males	Baseline test					Rehydration test				*p*_*interaction*_
	NW group (*n* = 8)	WS group 1 (*n* = 8)	WS group 2 (*n* = 8)	WS group 3 (*n* = 8)	Total (*n* = 32)	NW group (*n* = 8)	WS group 1 (*n* = 8)	WS group 2 (*n* = 8)	WS group 3 (*n* = 8)	
ICW (kg)	22.0 ± 2.6^b^	25.3 ± 4.5^b^	24.5 ± 2.3^b^	23.3 ± 1.5^b^	23.9 ± 3.1^b^	22.1 ± 2.6^b^	25.0 ± 4.6^b^	24.6 ± 2.3^b^	23.3 ± 1.3^b^	0.354
ICW/TBW (%)	62.8 ± 0.5^b^	62.8 ± 0.4^b^	62.8 ± 0.7^b^	62.8 ± 0.4^b^	62.8 ± 0.5^b^	62.7 ± 0.4^b^	62.7 ± 0.3^b^	62.7 ± 0.7^b^	62.7 ± 0.5^b^	0.960
ECW (kg)	13.1 ± 1.7^b^	15.0 ± 2.7^b^	14.7 ± 1.3^b^	13.8 ± 1.1^b^	14.1 ± 1.9^b^	13.2 ± 1.7^b^	14.9 ± 2.8^b^	14.6 ± 1.2^b^	13.9 ± 1.0^b^	0.397
ECW/TBW (%)	37.2 ± 0.5^b^	37.2 ± 0.4^b^	37.2 ± 0.7^b^	37.2 ± 0.4^b^	37.2 ± 0.5^b^	37.3 ± 0.4^b^	37.3 ± 0.3^b^	37.3 ± 0.7^b^	37.3 ± 0.5^b^	0.960
ECW/ICW (%)	59.4 ± 1.3^b^	59.2 ± 1.0^b^	59.2 ± 1.8^b^	59.3 ± 1.1^b^	59.3 ± 1.3^b^	59.6 ± 1.0^b^	59.5 ± 0.8^b^	59.4 ± 1.8^b^	59.6 ± 1.3^b^	0.956
TBW (kg)	35.1 ± 4.3^b^	40.3 ± 7.2^b^	39.5 ± 3.6^b^	37.2 ± 2.6^b^	38.0 ± 4.9^b^	35.2 ± 4.2^b^	39.9 ± 7.4^b^	39.2 ± 3.5^b^	37.2 ± 2.3^b^	0.355
TBW/BW (%)	55.0 ± 4.0^b^	60.4 ± 4.5^b^	56.5 ± 4.4^b^	58.9 ± 4.1^b^	57.7 ± 4.6^b^	54.7 ± 4.0^b^	59.7 ± 4.4^b^	56.2 ± 4.7^b^	59.3 ± 3.6^b^	0.224
TBW/FFM (%)	73.3 ± 0.2	73.4 ± 0.2	73.3 ± 0.4	73.3 ± 0.1	73.3 ± 0.2	74.7 ± 3.7	73.4 ± 0.2	73.3 ± 0.4	74.6 ± 3.7	0.585

**Females**	**NW group (n = 8)**	**WS group 1 (n = 8)**	**WS group 2** **(n = 8)**	**WS group 3** **(n = 8)**	**Total** **(n = 32)**	**NW group** **(n = 8)**	**WS group 1** **(n = 8)**	**WS group 2** **(n = 8)**	**WS group 3** **(n = 8)**	***P*_*interaction*_**

ICW (kg)	16.8 ± 1.9	17.4 ± 2.4	17.7 ± 1.8	18.9 ± 1.6	17.7 ± 2.0	16.2 ± 1.9	17.1 ± 2.4	17.5 ± 1.8	18.3 ± 1.7	0.088
ICW/TBW (%)	62.1 ± 0.5	62.1 ± 0.5	61.9 ± 0.4	62.0 ± 0.8	62.0 ± 0.5	61.9 ± 0.6	61.9 ± 0.4	61.8 ± 0.6	62.0 ± 0.7	0.625
ECW (kg)	10.2 ± 1.0*^a^*	10.7 ± 1.6*^a^*	10.9 ± 1.1	11.6 ± 1.1*^a^*	10.8 ± 1.3	10.0 ± 1.0	10.6 ± 1.6	10.8 ± 1.1	11.3 ± 1.1	**0.043**
ECW/TBW (%)	37.9 ± 0.5	37.9 ± 0.5	38.1 ± 0.4	38.0 ± 0.8	38.0 ± 0.5	38.1 ± 0.6	38.1 ± 0.4	38.2 ± 0.6	38.0 ± 0.7	0.625
ECW/ICW (%)	61.0 ± 1.3	61.2 ± 1.3	61.5 ± 1.0	61.3 ± 2.0	61.2 ± 1.4	61.5 ± 1.5	61.5 ± 1.1	61.8 ± 1.5	61.4 ± 1.9	0.621
TBW (kg)	27.0 ± 2.8*^a^*	28.1 ± 4.1*^a^*	28.5 ± 2.8	30.4 ± 2.7*^a^*	28.5 ± 3.3	26.2 ± 2.9	27.7 ± 4.0	28.3 ± 2.9	29.6 ± 2.8	**0.055**
TBW/BW (%)	49.2 ± 2.2	52.4 ± 3.6	51.5 ± 3.0	53.8 ± 4.4	51.7 ± 3.7	48.9 ± 2.8	51.6 ± 3.5	51.0 ± 3.1	52.4 ± 4.6	0.275
TBW/FFM (%)	74.5 ± 3.7	73.2 ± 0.2	73.2 ± 0.1	73.3 ± 0.2	73.6 ± 1.8	73.3 ± 0.1	73.1 ± 0.3	73.3 ± 0.2	73.3 ± 0.2	0.442

*Values are shown as the mean ± standard deviation (SD).*

*^a^p < 0.05 in the comparison between the baseline test and rehydration test.*

*^b^p < 0.05 in the comparison between men and women within a group. For men, no significant interactions between time and volume were found in ICW, ICW/TBW, ECW, ECW/TBW, ECW/ICW, TBW, TBW/BW, and TBW/FFM (all p > 0.05). For women, a significant interaction between time and volume was only found in ECW (F = 3.096, p = 0.043), not in ICW, ICW/TBW, ECW/TBW, ECW/ICW, TBW, TBW/BW, and TBW/FFM (all p > 0.05).*

For women, comparing the baseline and rehydration tests, no significant differences were found in ICW, ICW/TBW, ECW, ECW/TBW, ECW/ICW, TBW, TBW/BW, and TBW/FFM among the four groups (all *p* > 0.05). Moreover, the main effects of time were statistically significant in all of the body composition indices (all *p* < 0.01), but volumes were not (all *p* > 0.05). Meanwhile, a significant interaction between time and volume was only found in ECW (*p* = 0.043), and TBW tended toward significance (*p* = 0.055). Follow-up t-tests indicated that the reductions of ECW in WS group 1, WS group 3, and the NS group differed significantly (*p* = 0.028, *p* = 0.001, and *p* = 0.029, respectively), while no significant change was found in WS group 2 (*p* = 0.329) when comparing baseline and rehydration tests. Compared with NW group, there were no significant differences between WS group 1 (*p* = 1.000), WS group 2 (*p* = 1.000), and WS group 3 (*p* = 0.288). When comparing the d-value (ECW1-ECW2), no significant differences were found among the four groups (*p* = 0.909). Furthermore, TBW decreased significantly in WS group 1, WS group 3, and the NW group (*p* = 0.006, *p* = 0.001, and *p* = 0.010, respectively), with that of WS group 2 remaining unchanged (*p* = 0.169). These *t*-tests of the TBW should be interpreted cautiously, as the interaction was not significant. Compared with the NW group, there were no significant differences between WS group 1 (*p* = 1.000), WS group 2 (*p* = 1.000), and WS group 3 (*p* = 0.256).

Referring to the differences among men and women, in the baseline test, higher ICW, ECW, TBW, and TBW/BW and lower ICW/TBW, ECW/TBW, and ECW/ICW were found in men, compared to women (all *p* < 0.05), but no significant differences were found in TBW/FFM (*p* > 0.05). Furthermore, significant differences were found in all of the body composition indices in each group, both in the baseline and rehydration tests (all *p* < 0.05).

## Discussion

In this study, we aimed to explore the impacts of different amounts of water supplementation on the body composition indices of healthy young male and female adults after water restriction for 12 h, for the first time in China. The osmolality of the urine and plasma was measured to determine the hydration status of the participants: on one hand, to make sure that they were all dehydrated after water restriction for 12 h; on the other hand, to determine the effects of water supplementation on body composition under the improved hydration status. Contrary to our expectations, the results revealed that, although the volumes of water supplementation differed, healthy individuals may have acute deficits of TBW. In this study, thirst decreased and the hydration status improved significantly after participants drank water, and no significant differences were found between participants who drank 500 mL compared to 200 mL. Meanwhile, the thirst increased and the hydration status got worse (with the urine osmolality increasing) among those who drank no water. Thus, we may conclude that water supplementation could prevent the adverse effects of water restriction on thirst and urine osmolality and that the minimum amount of water to attenuate the adverse effects of water restriction on thirst and urine osmolality is 200 mL.

Previous research works exploring the influence of acute water supplementation on body composition have been inconsistent. For ICW, no significant differences were revealed in this study in adults, even with different amounts of water supplementation. In results reported from an experiment involving rats, an increase in ICW was observed in rats after water and NaCl consumption; this is essentially the inverse of the findings from our study in healthy young men and women (52). The differences in the type of fluid supplementation and the type of dehydration could explain such differences. In the abovementioned study, the rats were provided supplementation of water and NaCl while, in our study, participants consumed different amounts of purified water. Moreover, the rats had acute body fluid deficits, with up to 20% reduction in ECW and up to 2% reduction in ICW; however, the participants of our study only underwent 12 h of water restriction. Interestingly, we only observed significant reductions in ECW among women who had 500 or 100 mL of water supplementation, while that of those who had 200 mL of water did not decrease; meanwhile, the *post hoc* analysis reported no significant differences among the four groups, when compared with each other. In contrast, no significant changes were found among men after water supplementation. Therefore, it could be inferred that the ECW among women after 12 h water restriction still decreased without water supplementation, with concomitant increases in thirst and urine osmolality. Too little (100 ml) or much (500 ml) water supplementation did not prevent the adverse effects of water restriction on the ECW, but the volume of 200 ml did. Thus, 200 mL of water supplementation of 200 may be the minimum that could lead to an increase in ECW among young women. The optimal volume of water supplementation that increases the ECW may be between 200 ml and 500 ml among young women after 12 h of water restriction. More studies should be conducted to explore this issue. Our results showed that the hydration status of participants who drank 500 ml and 200 ml improved and did not differ significantly between groups. Hence, 200 ml of water could prevent the adverse effects on hydration status due to 12 h of water restriction, but including the body water content (e.g., ICW and ECW), it may be that the stability of the homeostasis of water in the human body contributed to these unexpected results.

Furthermore, women were more sensitive to water supplementation than men, which was consistent with other studies, with more aspects of body composition changed in women than that of men (31). A study conducted among 56 male wrestlers has reported that, after drinking a carbohydrate–electrolyte solution, the ECW significantly decreased incrementally from the pre-dehydration test to rehydration test; even after 2 hours of rehydration, the ECW did not return to the baseline level (53). Moreover, a study evaluating the effects of different kinds of water demonstrated no changes in ECW among 88 amateur male athletes after 300 mL of water supplementation, while that of those who drank mineral water decreased, with a concomitant increase in ICW (54). This indicates that the type of fluid may lead to the differences in the changes in body composition. Notwithstanding, as there exist few related studies considering the effects of water supplementation on body composition, it was difficult to compare the results of this study with those of other studies. In this study, even after 500 mL of water supplementation, ECW still decreased. These results may be attributed to the metabolism of water in the human body and the amount of water supplied to the participants after water restriction for 12 h. In our study, participants drank the water within 10 min and the interval of the rehydration test was 90 min after water supplementation, which was set according to the results of some studies and the pre-investigation for this study (55–57). Water may have an associated metabolic process, with many physiological reactions potentially occurring, among young adults after water supplementation. Considering the interval of this process, it may take longer than 90 min for the water to be absorbed and metabolized completely in the body. Furthermore, the results were pertinent to the debate over the dose-effect relationship of acute water supplementation on body composition, in which the amount of 500 mL of water did not have the same effect on body composition as 200 mL of water. Furthermore, although studies have shown the high validity of the BIA in measuring body composition indices, some studies arrived at an opposite conclusion, reporting the inaccuracy of the BIA (58). Maybe such a lack of validity of the BIA could have contributed to the minor differences in ECW among young women. Studies have also shown that the BIA was only valid in measuring the body composition among obese people or people with good hydration status. Furthermore, it has been shown that critical factors, such as variations in machine specifications, technical skill, the subject, and environmental factors, have an impact on the BIA results (59).

In this study, we detailed a preliminary exploration of the effects of different volumes of water on body composition indices after 12 h of water restriction. In future, more related studies should be implemented to explore this issue using the gold-standard techniques, such as isotope dilution (60), or still using BIA but among different people with different ages. The current consensus is that, in free-living conditions, no matter the amount of water that healthy people consume, the total body water remains stable. In this study, the consumption of 500, 200, and 100 mL of water may have impacted the TBW, but such results were not detected in this study due to the sensitivity of the BIA, in contrast to the results of a previous study (61). Studies have shown that the sensitivity is not only related to the volume of fluids, but also to the resistivity. The results of the study indicated that the resistivity of the ECW and ICW was different and associated with the types of the fluid, for which the resistivity of ICW was approximately twice that of ECW and increased slightly (but not significantly) following infusion; meanwhile, the resistivity of ECW was significantly decreased by NaCl infusion in rats (62). Furthermore, it may be that acute water supplementation did not influence the TBW, indicating that further studies, using other methods to measure the body composition and larger volumes of water provided to the participants after water supplementation, are needed. Interestingly, the TBW decreased in women with water supplementation of 500 and 100 mL and no water supplementation, while no decrease was found among those with 200 mL of water. Perhaps amounts of water between 200 and 500 mL could improve the TBW among young women after 12 h of water restriction. Data from studies exploring the effect of water supplementation on TBW have been inconsistent. Similar to our study, some studies have not observed any beneficial effects of water supplementation on TBW. A study conducted among 13 active men has reported that the TBW was unaffected after consuming up to 591 mL of water (63). Furthermore, among 45 healthy male and female runners, the TBW remained unchanged after water replenishment of 538 mL and 533 mL, respectively (64). In a study investigating the effects of carbohydrate–electrolyte fluid on body composition among young healthy athletes, it was found that, during the 2 h of rehydration, no significant changes in TBW were observed (49). The results of a randomized study revealed that, after drinking 400 mL fluid, no significant differences in TBW were found among adults after fasting for 10 h (65). In addition, the TBW even decreased from baseline among 10 young men and women when measured 150 min after administration with 466 mL of deionized water (66). Contrary to the results of the studies, the beneficial impacts of water supplementation on TBW have been observed in some research works. One study investigating the effects of different volumes of water on TBW has revealed that, after 500 mL of water supplementation, an increase in TBW by 0.21 kg was found in men, but not in women (67). Similarly, another study has shown that participants who consumed 1,000 mL of water had higher TBW than their counterparts who drank nothing (31). The intervals used for measuring the changes in the TBW, as well as the types of fluids supplied to the participants, could explain the differences in the abovementioned studies.

Studies have demonstrated that body composition differs as a result of many factors, such as age, gender, BMI, nutrition status, and physiological stages (68, 69). Even for the same subjects, inter-daily variabilities of body composition have also been observed (70). Our data indicated differences in all of the considered indices, including ICW, ECW, TBW, ICW/TBW, ECW/TBW, and TBW/BW, between all the men and women. Consistent with a study conducted among young adults under free-living conditions, many aspects of body composition, including ICW, ECW, and TBW, differed significantly between men and women (20, 27, 71). One study conducted among children has confirmed similar differences between genders, showing that girls have lower TBW and TBW/BW than boys (26). The results of our study indicated that differences between men and women exist, even with different hydration statuses.

This research had some strengths. First, it provides the first exploration considering whether acute water supplementation has an impact on body composition among healthy young men and women after water restriction for 12 h. In addition, a randomized controlled design was conducted among young male and female adults, to reduce the related bias induced by gender. Second, the hydration status of the participants was measured through urine osmolality and the investigators observed the lips of the participants at all times, to make sure that they followed the study procedure. Furthermore, in this study, the Inbody 720 was highly associated with DXA, with the correlation coefficient reaching 0.98. Furthermore, the result of each measurement was printed and calculated by the machine, following the 4C model. However, this study had certain limitations. For example, studies have shown a relationship between body hydration conditions, body composition, and microbiota (72), but no related biomarkers were measured in our study. Furthermore, more plasma biomarkers, including copeptin, were not explored in this study. Finally, the tiny changes in certain body composition indices, including the ECW, ICW, and TBW, may not be well-measured by the BIA. Therefore, future studies should use the BIA combined with gold-standard techniques (e.g., isotope dilution), to examine the effects of water supplementation on body composition.

## Conclusion

Water supplementation has been shown to redistribute the water content in the body, with no decrease in ECW. Our results suggest that 200 mL of water may be the minimum volume to ensure a better distribution of the body water content among young female adults—but not men—after water restriction for 12 h. It may be that the sensitivity of the BIA did not allow for the detection of the tiny changes in certain body composition indices. More studies using other methods to measure these changes in body composition indices should be conducted, to address this issue.

## Data Availability Statement

The raw data supporting the conclusions of this article will be available from the corresponding author upon reasonable request.

## Ethics Statement

The study protocol was approved by the Peking University Institutional Review Board. The ethical approval project identification code was IRB00001052-16071. The study was conducted according to the guidelines of the Declaration of Helsinki. All subjects signed the informed consent form before participating in the study.

## Author Contributions

JZ was responsible for the implementation of the study and drafting of the article. GM was responsible for the design of the study and provided substantive revisions to the initial draft of the article. NZ, SD, and GM were responsible for the design of the study, quality management, and control of the implementation of the study. JZ and SL were responsible for the recruitment of participants. JZ, NZ, and SL were responsible for the implementation of the study. All authors were involved in the revision of the manuscript and approved this final version.

## Conflict of Interest

The authors declare that the research was conducted in the absence of any commercial or financial relationships that could be construed as a potential conflict of interest.

## Publisher’s Note

All claims expressed in this article are solely those of the authors and do not necessarily represent those of their affiliated organizations, or those of the publisher, the editors and the reviewers. Any product that may be evaluated in this article, or claim that may be made by its manufacturer, is not guaranteed or endorsed by the publisher.

## Abbreviations

TWI: Total water intake
TBW: Total body water
ICW: Intracellular water
ECW: Extracellular water
BW: Body weight.
