# Supplementary material for: Acute Water Supplementation Improved the Body Composition of Young Female Adults After Water Restriction of 12 h in Baoding, China: A Randomized Controlled Trial (RCT)
Source: Front Nutr. 2022 Jun 20;9:880630. doi: 10.3389/fnut.2022.880630 (PMC9251362; doi:10.3389/fnut.2022.880630)
Supplement: Supplementary file 1 [file Table_1.docx]

**Table S1** The characteristics of participants

|  | | Baseline test | | | |  |  |  | Rehydration test | | | |  | |
| --- | --- | --- | --- | --- | --- | --- | --- | --- | --- | --- | --- | --- | --- | --- |
|  | WS group 1 (n=16) | WS group 2 (n=16) | WS group 3 (n=16) | NW group (n=16) | *F* | *P* |  | WS group 1 (n=16) | WS group 2 (n=16) | WS group 3 (n=16) | NW group (n=16) | *F* | *P* |  |
| Age（year） | | 21.3±1.4 | 20.8±1.0 | 20.9±0.9 | 21.1±0.8 | 0.858 | 0.468 |  | 21.3±1.4 | 20.8±1.0 | 20.9±0.9 | 21.1±0.8 | 0.858 | 0.468 |
| Height (cm) | | 166.5±9.3 | 167.1±8.7 | 167.6±7.2 | 162.2±7.5 | 1.445 | 0.239 |  | 166.5±9.3 | 167.1±8.7 | 167.6±7.2 | 162.2±7.5 | 1.445 | 0.239 |
| Weight (kg)* | | 60.5±12.6 | 63.0±11.5 | 59.9±5.1 | 59.2±10.4 | 0.404 | 0.750 |  | 60.5±12.7 | 62.9±11.4 | 59.8±5.2 | 59.1±10.4 | 0.421 | 0.738 |
| BMI (kg/m^2^)* | | 21.6±3.0 | 22.5±3.4 | 21.4±1.6 | 22.4±3.0 | 0.679 | 0.568 |  | 21.6±3.0 | 21.3±1.5 | 22.4±3.0 | 22.5±3.4 | 0.661 | 0.579 |
| Systolic pressure (mmHg)* | | 109±11 | 106±9 | 103±10 | 106±11 | 0.667 | 0.576 |  | 110±10 | 106±7 | 104±11 | 103±11 | 1.277 | 0.290 |
| Diastolic pressure (mmHg)* | | 65±7 | 63±8 | 62±7 | 66±11 | 1.013 | 0.393 |  | 65±6 | 60±7 | 62±8 | 63±11 | 1.271 | 0.292 |

**Note**: *, There were significant differences between the baseline test and dehydration test; Values are shown as the mean ± standard deviation (SD).

**Table S2** The thirst, urine and plasma biomarkers of participants

|  | Baseline test | | | | *F* | *P* | Rehydration test | | | | Interaction | |
| --- | --- | --- | --- | --- | --- | --- | --- | --- | --- | --- | --- | --- |
|  | WS group 1  (n=16) | WS group 2  (n=16) | WS group 3  (n=16) | NW group  (n=16) |  |  | WS group 1  (n=16) | WS group 2 (n=16) | WS group 3 (n=16) | NW group (n=16) | *F* | *P* |
| Thirst | 5.5±1.3 | 5.2±2.2 | 5.7±2.1 | 5.1±2.0 | 0.339 | 0.797 | 3.6±1.7^#†^ | 4.2±1.9^†^ | 5.4±2.0^bd^ | 6.3±1.2^†ce^ | 6.172 | 0.001 |
| **Plasma biomarkers** | | |  |  |  |  |  |  |  |  |  |  |
| Osmolality (mOsm/kg) | 290±5 | 290±6 | 291±4 | 291±6 | 0.190 | 0.903 | 288±6 | 289±7 | 291±4 | 290±5 | 2.314 | 0.085 |
| Glucose (mmol/L) | 4.5±0.3 | 4.7±0.6 | 4.5±0.3 | 4.5±0.4 | 0.493 | 0.688 | 4.7±0.4 | 4.9±0.6 | 4.8±0.5 | 4.8±0.4 | 0.133 | 0.940 |
| Na (mmol/L) | 140±2 | 139±3 | 140±4 | 140±3 | 0.560 | 0.644 | 138±1 | 139±3 | 139±3 | 139±2 | 0.632 | 0.597 |
| K (mmol/L) | 4.2±0.4 | 4.2±0.4 | 4.3±0.5 | 4.4±0.4 | 0.499 | 0.684 | 4.3±0.4 | 4.3±0.5 | 4.3±0.5 | 4.4±0.3 | 0.326 | 0.807 |
| Cl (mmol/L) | 104±2 | 104±1 | 104±2 | 105±2 | 1.748 | 0.167 | 103±2 | 104±2 | 104±3 | 104±1 | 0.437 | 0.727 |
| Ca (mmol/L) | 2.40±0.07 | 2.39±0.08 | 2.40±0.13 | 2.41±0.10 | 0.252 | 0.860 | 2.42±0.06 | 2.42±0.07 | 2.41±0.12 | 2.42±0.08 | 0.511 | 0.676 |
| Phosphorus (mmol/L) | 1.31±0.16 | 1.25±0.15 | 1.32±0.16 | 1.29±0.11 | 0.707 | 0.552 | 1.18±0.12 | 1.24±0.21 | 1.21±0.16 | 1.16±0.12 | 1.675 | 0.182 |
| Mg (mmol/L) | 0.89±0.05 | 0.87±0.05 | 0.86±0.05 | 0.86±0.06 | 0.850 | 0.472 | 0.88±0.04 | 0.86±0.06 | 0.85±0.06 | 0.86±0.06 | 0.020 | 0.985 |
| Creatine (mmol/L) | 64±15 | 65±14 | 66±13 | 61±12 | 0.441 | 0.725 | 62±15 | 64±13 | 66±14 | 59±11 | 0.599 | 0.618 |
| Nitrogen (mmol/L) | 4.28±1.19 | 4.07±0.91 | 4.46±1.23 | 4.53±1.14 | 0.528 | 0.665 | 3.80±1.08 | 3.86±0.76 | 4.28±1.09 | 4.34±1.04 | 1.342 | 0.269 |
| **Urinary Biomarkers** | | | | |  |  |  |  |  |  |  |  |
| Osmolality (mOsm/kg) | 814±221 | 833±151 | 820±189 | 776±134 | 0.305 | 0.822 | 353±200^†^ | 571±237^†a^ | 787±231^bd^ | 935±125^†cef^ | 20.129 | <0.001 |
| Volume | 270±82 | 278±97 | 308±143 | 298±83 | 0.444 | 0.723 | 334±186 | 203±90 | 166±85 | 145±80 | 8.430 | <0.001 |
| Na (mmol/L) | 162±68 | 184±67 | 187±59 | 170±34 | 0.636 | 0.594 | 66±47 | 110±66 | 167±84^bd^ | 164±64^ce^ | 5.635 | 0.002 |
| K (mmol/L) | 38.6±13.4 | 43.1±15.0 | 38.6±12.9 | 37.8±26.4 | 0.288 | 0.834 | 22.3±10.8 | 35.8±14.4 | 58.3±25.8^bd^ | 60.1±26.8^ce^ | 13.532 | <0.001 |
| Cl (mmol/L) | 150±44 | 161±55 | 168±52 | 151±30 | 0.549 | 0.651 | 79±50 | 131±71^a^ | 201±88^bd^ | 200±67^ce^ | 9.178 | <0.001 |
| Ca (mmol/L) | 1.99±1.20 | 2.44±1.62 | 2.98±1.80 | 2.69±1.16 | 1.299 | 0.283 | 0.88±0.70 | 1.85±1.56 | 2.41±1.60 | 2.93±1.59 | 2.304 | 0.086 |
| Phosphorus (mmol/L) | 32.73±15.21 | 37.55±14.26 | 37.38±14.84 | 28.84±15.35 | 1.249 | 0.300 | 9.07±7.32 | 10.89±4.32 | 19.33±7.97^bd^ | 23.37±9.30^ce^ | 5.246 | 0.003 |
| Mg (mmol/L) | 2.74±1.06 | 3.27±1.39 | 4.10±2.32 | 2.82±1.06 | 2.615 | 0.059 | 1.25±0.79 | 2.58±2.02 | 3.22±1.69 | 3.00±1.91 | 1.867 | 0.145 |
| pH | 6.3±0.4 | 6.5±0.4 | 6.4±0.3 | 6.6±0.5 | 1.924 | 0.135 | 6.2±0.4 | 6.3±0.4 | 6.3±0.3 | 6.2±0.4 | 1.677 | 0.181 |
| USG | 1.024±0.006 | 1.024±0.006 | 1.023±0.006 | 1.022±0.006 | 0.404 | 0.751 | 1.013±0.008 | 1.018±0.006 | 1.023±0.007^bd^ | 1.027±0.006^ce^ | 10.822 | <0.001 |
| Hydration statuses (%) | | |  |  |  |  |  |  |  |  |  |  |
| baseline | 9 (56.3) | 10 (62.5) | 11 (68.8) | 7 (43.7) | 10.891^Φ^ | 0.092 | 0 (0.0)^†^ | 5 (31.2)^†^ | 10 (62.5) | 14 (87.5)^†^ | 35.359 | <0.001 |
| Middle hydration | 5 (31.2) | 6 (37.5) | 2 (12.5) | 9 (56.3) |  |  | 5 (31.2) | 3 (18.8) | 4 (25.0) | 2 (12.5) |  |  |
| Optimal hydration | 2 (12.5) | 0 (0.0) | 3 (18.7) | 0 (0.0) |  |  | 11 (68.8) | 8 (50.0) | 2 (12.5) | 0 (0.0) |  |  |

**Note**: Values are shown as the mean ± standard deviation (SD), with the exception that percentages were shown as n (percentage); *, There were significant differences among the four groups during the baseline test; ^#^, there were significant differences among the four groups during the rehydration test; ^†^, there were significant differences between baseline test and rehydration test within the group. ^Φ^,the statistical value was χ2 of Chi-square. a, significant differences were found between WS group 1 with WS group 2; b, significant differences were found between WS group 1 with WS group 3; c, significant differences were found between WS group 1 with NW group; d, significant differences were found between WS group 2 with WS group 3; e, significant differences were found between WS group 2 with NW group; f, significant differences were found between WS group 3 with NW group.

In thirst, comparing with baseline test, the scores of thirst decreased in WS group 1, WS group 2 (*t*=3.576, *p*=0.003; *t*=2.242, *p*=0.042), and there was no significant change was found in WS group 3 (*t*=0.645, *p*=0.529), but a significant increase was found in NW group (*t*=-2.267, *p*=0.039), during rehydration test. Comparing WS group 1 with WS group 2, no significant difference was found in the thirst in rehydration test (*p=*0.367). In the osmolality of urine, significant reductions were found in WS group 1, WS group 2 (*t*=7.576, *p*＜0.001; *t*=3.393, *p*=0.004) and no significant difference was found in WS group 3 (*t*=0.542, *p*=0.596), but a significant increase was found in NW group (*t*=-4.728, *p*＜0.001), during rehydration test. In hydration status, comparing with baseline test, the hydration status were better in WS group 1, WS group 2 (χ2=16.154, *p*＜0.001; χ2=11.130, *p*=0.004), and no significant difference was found in WS group 3 (χ2=0.977, *p*=0.765), but the hydration status was getting worse in NW group (χ2=6.788, *p*=0.023), in rehydration test. There was no significant difference between WS group 1 and WS group 2 in hydration status in rehydration test (χ2=5.841, *p*=0.090).

**Table S3.** The body composition of participants

|  | Baseline test | | | | | Rehydration test | | | | *p_interaction_* |
| --- | --- | --- | --- | --- | --- | --- | --- | --- | --- | --- |
| **Total** | | NW group  (n=16) | WS group 1  (n=16) | WS group 2 (n=16) | WS group 3 (n=16) | NW group (n=16) | WS group 1 (n=16) | WS group 2 (n=16) | WS group 3 (n=16) |  |
| ICW | 19.4±3.5 | | 21.4±5.3 | 21.2±4.2 | 21.1±2.8 | 19.1±3.7 | 21.1±5.4 | 21.0±4.2 | 20.8±3.7 | 0.921 |
| ICW/TBW (%) | 62.5±0.6 | | 62.4±0.6 | 62.4±0.7 | 62.4±0.7 | 62.3±0.6 | 62.3±0.5 | 62.3±0.7 | 62.3±0.7 | 0.796 |
| ECW | 11.7±2.0 | | 12.8±3.1 | 12.8±2.3 | 12.7±1.6 | 11.6±2.1 | 12.7±3.1 | 12.7±2.3 | 12.6±1.7 | 0.909 |
| ECW/TBW (%) | 37.6±0.6 | | 37.6±.6 | 37.6±0.7 | 37.6±0.7 | 37.7±0.6 | 37.7±0.5 | 37.7±0.8 | 37.7±0.7 | 0.796 |
| ECW/ICW (%) | 60.2±1.5 | | 60.2±1.5 | 60.3±1.8 | 60.3±1.8 | 60.6±1.6 | 60.5±14 | 60.6±2.0 | 60.5±1.8 | 0.801 |
| TBW | 31.1±5.5 | | 34.2±8.5 | 34.0±6.4 | 33.8±4.3 | 30.7±5.8 | 33.8±8.5 | 33.7±6.4 | 33.4±4.7 | 0.918 |
| TBW/BW (%) | 52.1±4.3 | | 56.4±5.7 | 54.0±4.5 | 56.3±4.9 | 51.8±4.5 | 55.7±5.7 | 53.6±4.7 | 55.8±5.3 | 0.643 |
| TBW/FFM (%) | 73.9±2.6 | | 73.3±0.2 | 73.3±0.3 | 73.3±0.2 | 74.0±2.6 | 73.3±0.2 | 73.3±0.3 | 74.0±2.6 | 0.819 |

**Note**: Values are shown as the mean ± standard deviation (SD). ^a^*p*<0.05 in comparison between the baseline test and rehydration test. ^b^*p*<0.05 in comparison between males and females within group. No significant differences were found in ICW, ICW/TBW, ECW, ECW/TBW, ECW/ICW and TBW (*F*=0.813, *p*=0.492; *F*=0.042, *p*=0.988; *F*=0.916, *p*=0.439; *F*=0.042, *p*=0.988; *F*=0.046, *p*=0.987; *F*=0.854, *p*=0.470), and significant difference was found in TBW/BW (*F*=2.928, *p*=0.041) among the four groups in the baseline test; in rehydration test, no significant differences were found in ICW, ICW/TBW, ECW, ECW/TBW, ECW/ICW, TBW and TBW/BW among the four groups (*F*=0.775, *p*=0.512; *F*=0.014, *p*=0.998; *F*=0.860, *p*=0.467; *F*=0.014, *p*=0.998; *F*=0.016, *p*=0.997; *F*=0.809, *p*=0.494; *F*=2.264, *p*=0.090). When comparing rehydration test with baseline test, no significant interactions between TIME×VOLUME were found in ICW, ICW/TBW, ECW, ECW/TBW, ECW/ICW, TBW, TBW/BW and TBW/FFM (*F*=0.163, *p*=0.921; *F*=0.341, *p*=0.796; *F*=0.181, *p*=0.909; *F*=0.341, *p*=0.796; *F*=0.333, *p*=0.801; *F*=0.168, *p*=0.918; *F*=0.560, *p*=0.643; *F*=0.348, *p*=0.558). Significant main effects of time were found in ICW, ICW/TBW, ECW, ECW/TBW, ECW/ICW, TBW and TBW/BW (*F*=25.892, *p*<0.001; *F*=17.939, *p*<0.001; *F*=10.858, *p*=0.002; *F*=17.939, *p*<0.001; *F*=17.754, *p*<0.001; *F*=20.477, *p*<0.001; *F*=11.219, *p*=0.001), but no significant difference found in TBW/FFM (*F*=1.348, *p*=0.267). No significant main effects of volume were found in ICW, ICW/TBW, ECW, ECW/TBW, ECW/ICW, TBW, TBW/BW and TBW/FFM (*F*=0.795, *p*=0.501; *F*=0.019, *p*=0.996; *F*=0.889, *p*=0.452; *F*=0.019, *p*=0.996; *F*=0.022, *p*=0.996; *F*=0.832, *p*=0.481; *F*=2.609, *p*=0.060; *F*=1.348, *p*=267).

**Table S4.** The body composition of males and females

|  | Baseline test | | | | | Rehydration test | | | | *p_interaction_* |
| --- | --- | --- | --- | --- | --- | --- | --- | --- | --- | --- |
| **Males** | NW group (n=8) | WS group 1 (n=8) | WS group 2  (n=8) | WS group 3  (n=8) | Total  (n=32) | NW group  (n=8) | WS group 1  (n=8) | WS group 2  (n=8) | WS group 3  (n=8) |  |
| ICW (kg) | 22.0±2.6^b^ | 25.3±4.5^b^ | 24.5±2.3^b^ | 23.3±1.5^b^ | 23.9±3.1^b^ | 22.1±2.6^b^ | 25.0±4.6^b^ | 24.6±2.3^b^ | 23.3±1.3^b^ | 0.354 |
| ICW/TBW (%) | 62.8±0.5^b^ | 62.8±0.4^b^ | 62.8±0.7^b^ | 62.8±0.4^b^ | 62.8±0.5^b^ | 62.7±0.4^b^ | 62.7±0.3^b^ | 62.7±0.7^b^ | 62.7±0.5^b^ | 0.960 |
| ECW (kg) | 13.1±1.7^b^ | 15.0±2.7^b^ | 14.7±1.3^b^ | 13.8±1.1^b^ | 14.1±1.9^b^ | 13.2±1.7^b^ | 14.9±2.8^b^ | 14.6±1.2^b^ | 13.9±1.0^b^ | 0.397 |
| ECW/TBW (%) | 37.2±0.5^b^ | 37.2±0.4^b^ | 37.2±0.7^b^ | 37.2±0.4^b^ | 37.2±0.5^b^ | 37.3±0.4^b^ | 37.3±0.3^b^ | 37.3±0.7^b^ | 37.3±0.5^b^ | 0.960 |
| ECW/ICW (%) | 59.4±1.3^b^ | 59.2±1.0^b^ | 59.2±1.8^b^ | 59.3±1.1^b^ | 59.3±1.3^b^ | 59.6±1.0^b^ | 59.5±0.8^b^ | 59.4±1.8^b^ | 59.6±1.3^b^ | 0.956 |
| TBW (kg) | 35.1±4.3^b^ | 40.3±7.2^b^ | 39.5±3.6^b^ | 37.2±2.6^b^ | 38.0±4.9^b^ | 35.2±4.2^b^ | 39.9±7.4^b^ | 39.2±3.5^b^ | 37.2±2.3^b^ | 0.355 |
| TBW/BW (%) | 55.0±4.0^b^ | 60.4±4.5^b^ | 56.5±4.4^b^ | 58.9±4.1^b^ | 57.7±4.6^b^ | 54.7±4.0^b^ | 59.7±4.4^b^ | 56.2±4.7^b^ | 59.3±3.6^b^ | 0.224 |
| TBW/FFM (%) | 73.3±0.2 | 73.4±0.2 | 73.3±0.4 | 73.3±0.1 | 73.3±0.2 | 74.7±3.7 | 73.4±0.2 | 73.3±0.4 | 74.6±3.7 | 0.585 |
| **Females** | NW group (n=8) | WS group 1 (n=8) | WS group 2  (n=8) | WS group 3  (n=8) | Total  (n=32) | NW group  (n=8) | WS group 1  (n=8) | WS group 2  (n=8) | WS group 3  (n=8) | *P_interaction_* |
| ICW (kg) | 16.8±1.9 | 17.4±2.4 | 17.7±1.8 | 18.9±1.6 | 17.7±2.0 | 16.2±1.9 | 17.1±2.4 | 17.5±1.8 | 18.3±1.7 | 0.088 |
| ICW/TBW (%) | 62.1±0.5 | 62.1±0.5 | 61.9±0.4 | 62.0±0.8 | 62.0±0.5 | 61.9±0.6 | 61.9±0.4 | 61.8±0.6 | 62.0±0.7 | 0.625 |
| ECW (kg) | 10.2±1.0^a^ | 10.7±1.6^a^ | 10.9±1.1 | 11.6±1.1^a^ | 10.8±1.3 | 10.0±1.0 | 10.6±1.6 | 10.8±1.1 | 11.3±1.1 | **0.043** |
| ECW/TBW (%) | 37.9±0.5 | 37.9±0.5 | 38.1±0.4 | 38.0±0.8 | 38.0±0.5 | 38.1±0.6 | 38.1±0.4 | 38.2±0.6 | 38.0±0.7 | 0.625 |
| ECW/ICW (%) | 61.0±1.3 | 61.2±1.3 | 61.5±1.0 | 61.3±2.0 | 61.2±1.4 | 61.5±1.5 | 61.5±1.1 | 61.8±1.5 | 61.4±1.9 | 0.621 |
| TBW (kg) | 27.0±2.8^a^ | 28.1±4.1^a^ | 28.5±2.8 | 30.4±2.7^a^ | 28.5±3.3 | 26.2±2.9 | 27.7±4.0 | 28.3±2.9 | 29.6±2.8 | **0.055** |
| TBW/BW (%) | 49.2±2.2 | 52.4±3.6 | 51.5±3.0 | 53.8±4.4 | 51.7±3.7 | 48.9±2.8 | 51.6±3.5 | 51.0±3.1 | 52.4±4.6 | 0.275 |
| TBW/FFM (%) | 74.5±3.7 | 73.2±0.2 | 73.2±0.1 | 73.3±0.2 | 73.6±1.8 | 73.3±0.1 | 73.1±0.3 | 73.3±0.2 | 73.3±0.2 | 0.442 |

**Note:** Values are shown as the mean ± standard deviation (SD). ^a^*p*<0.05 in comparison between the baseline test and rehydration test. ^b^*p*<0.05 in comparison between males and females within group. For males, when comparing the baseline test with rehydration test, significant main effects of time were found in ICW/TBW, ECW/TBW and ECW/ICW (*F*=11.047, *p*=0.002; *F*=11.047, *p*=0.002; *F*=11.100, *p*=0.002), but not in the ICW, ECW, TBW, TBW/BW and TBW/FFM (*F*=2.979, *p*=0.095; *F*=0.146, *p*=0.706; *F*=1.560, *p*=0.222; *F*=1.579, *p*=0.219; *F*=2.136, *p*=0.155); the main effects of volume were not statistically significant in ICW, ICW/TBW, ECW, ECW/TBW, ECW/ICW, TBW, TBW/BW and TBW/FFM (*F*=1.816, *p*=0.167; *F*=0.028, *p*=0.994; *F*=1.570, *p*=0.219; *F*=0.028, *p*=0.994; *F*=0.025, *p*=0.994; *F*=1.713, *p*=0.183; *F*=2.660, *p*=0.067; *F*=1.031, *p*=0.394); no significant interactions between time and volume were found in ICW, ICW/TBW, ECW, ECW/TBW, ECW/ICW, TBW, TBW/BW and TBW/FFM (*F*=1.130, *p*=0.354; *F*=0.099, *p*=0.960; *F*=1.025, *p*=0.397; *F*=0.099, p=0.960; *F*=0.105, *p*=0.956; *F*=1.127, *p*=0.355; *F*=1.549, *p*=0.224; *F*=0.657, *p*=0.585). For females, when comparing the baseline test with rehydration test, significant main effects of time were found in ICW, ICW/TBW, ECW, ECW/TBW, ECW/ICW, TBW and TBW/BW (*F*=49.934, *p*<0.001; *F*=7.874, *p*=0.009; *F*=33.563, *p*<0.001; *F*=7.874, *p*=0.009; *F*=7.858, *p*=0.009; *F*=48.184, p<0.001; *F*=12.950, *p*=0.001), but not in the TBW/FFM (*F*=1.090, *p*=0.305); no main effects of volume were not statistically significant in ICW, ICW/TBW, ECW, ECW/TBW, ECW/ICW, TBW, TBW/BW and TBW/FFM (*F*=1.568, *p*=0.219; *F*=0.118, *p*=0.949; *F*=1.553, *p*=0.223; *F*=0.118, p=0.949; *F*=0.116, *p*=0.950; *F*=1.578, *p*=0.217; *F*=1.966, *p*=0.137; *F*=1.031, *p*=0.394); significant interaction between time and volume was only found in ECW (*F*=3.096, *p*=0.043), not in ICW, ICW/TBW, ECW/TBW, ECW/ICW, TBW, TBW/BW and TBW/FFM (*F*=2.408, *p*=0.088; *F*=0.593, *p*=0.625; *F*=0.593, *p*=0.625; *F*=0.599, *p*=0.621; *F*=2.862, *p*=0.055; *F*=1.362, *p*=0.275; *F*=0.924, *p*=0.442).
